# Association between rapid and sustained remission and clinician- and patient-reported outcomes in patients with rheumatoid arthritis: post hoc analysis of data from the SELECT-COMPARE study

**DOI:** 10.1186/s13075-025-03580-1

**Published:** 2025-06-13

**Authors:** Laure Gossec, Jayesh Patel, Aditi Kadakia, Siran Fang, Yi Peng, Sander Strengholt, Peter C. Taylor, Andrew Östör

**Affiliations:** 1 Sorbonne Université, INSERM, Institut Pierre Louis d’Epidémiologie et de Santé Publique, Team Pepites, Paris, France; 2 Rheumatology Department, AP-HP, Pitié-Salpêtrière Hospital, Paris, France; 3 AbbVie Inc, North Chicago, IL USA; 4 Botnar Research Centre, University of Oxford, Oxford, UK; 5 Monash University and Emeritus Research Melbourne & ANU, Canberra, Australia

**Keywords:** Rheumatoid arthritis, Upadacitinib, Adalimumab, Patient-reported outcomes, Remission

## Abstract

**Background:**

Rapid remission has been shown to be beneficial in patients with early rheumatoid arthritis (RA). This study assessed the association of rapid and sustained remission with long-term clinician- and patient-reported outcomes (CRO/PROs) in patients treated with b/tsDMARDs.

**Methods:**

This post hoc analysis used pooled data on patients with moderately-to-severely active RA receiving upadacitinib or adalimumab from the SELECT-COMPARE trial (NCT02629159) and its open-label long-term extension (up to 5 years). This study assessed the effect of achieving rapid remission, time to remission, and time in sustained remission on CRO/PROs. Rapid remission was defined as a Disease Activity Score 28 with C-reactive protein (DAS28-CRP) < 2.6 after 12 weeks’ treatment. The outcomes of interest included a variety of PROs, such as pain, fatigue, quality of life, and CROs (28 swollen/tender joint counts). Where available, outcomes were assessed for up to 5 years; mean change in outcomes, as well as adjusted odds ratios (aOR) of achieving minimal clinically important differences (MCIDs) or normative values. Multivariate regression analyses were conducted adjusting for baseline covariates.

**Results:**

In total, 28% of patients (n/*N* = 247/865; mean disease duration: 8.2 ± 7.8 years) achieved rapid remission. Rapid remission was associated with significantly greater improvements from baseline in all outcomes at Week 26 and significantly greater odds of achieving MCIDs (aOR range: 2.2–5.6) or normative values (aOR range: 1.6–9.8) in most PROs, including pain, fatigue, and physical functioning, over the variable 5-year follow-up; significantly lower swollen/tender joint counts were also observed. Time to achieve remission was associated with better outcomes: for every month delay in achieving remission, likelihood of achieving MCIDs or normative values decreased, on average, by 13%. Increasing time spent in sustained remission was associated with long-term improvement in CRO/PROs.

**Conclusions:**

Remission is a key outcome in RA; this study showed that achieving rapid remission, as well as reducing time to achieving remission, was associated with less pain and fatigue, and better physical functioning and quality of life over 5 years. Similarly, increasing time spent in sustained remission correlated with sustained improvement in CRO/PROs. Striving for rapid, sustained remission leads to long-term benefits.

**Supplementary Information:**

The online version contains supplementary material available at 10.1186/s13075-025-03580-1.

## Introduction

Rheumatoid arthritis (RA) is a chronic immune-mediated inflammatory arthritis and is the most common form of inflammatory polyarthritis, estimated to affect approximately 18 million people worldwide [1]. RA has been shown to have a negative impact on daily activities and is associated with high disease burden and impaired health-related quality of life (HRQoL) [2, 3]. Moreover, RA is associated with significant economic burden, especially among patients who do not achieve disease control on treatment [4].

Treatment guidelines for RA recommend a treatment target of sustained remission or low disease activity in every patient [5]. Achieving remission has been associated with significant impacts on patient outcomes, including improved long-term clinical outcomes, better HRQoL, and increased productivity [4] Altogether, these benefits are likely to translate to reduced economic burden due to lower healthcare resource utilization, reduced healthcare costs, and increased work and activity abilities [4].

Currently, there is evidence indicating that achieving rapid remission is associated with clinical and functional benefits earlier in the disease course for RA patients using conventional synthetic (cs) disease-modifying antirheumatic drugs (DMARD) [6, 7]. Methotrexate (MTX), as csDMARD, is recommended as a first-line treatment, but rates of remission with MTX monotherapy are relatively low (and even if achieved, often not sustained), especially for patients with a longer disease duration [5]. However, there is some evidence that suggests that early, intensive treatment with a combination of MTX and a biologic or targeted synthetic (b/ts)DMARD may help patients achieve low disease activity or remission earlier [6].

The current study aims to further add to the evidence that rapid response to conventional [6, 7] and targeted therapy [8, 9] in patients in the established phase of disease is an indicator of better long-term outcomes, with respect to both disease activity measures and patient-reported outcomes (PROs). Most importantly, this study sought to evaluate the impact of the time to achieving remission, and the time spent in remission (herein referred to as sustained remission), on long-term PROs and clinician-reported outcomes (CROs). This is important because it has been shown that those patients who do not achieve rapid remission on advanced therapies are unlikely to achieve sustained remission later [7]. The three main objectives of this analysis were: (1) to assess the association of achieving rapid remission with long-term CRO/PROs in patients with RA receiving advanced therapies, (2) to evaluate the association of time to achieving remission with CRO/PROs, and (3) to assess the association of time in sustained remission with CRO/PROs.

## Materials and methods

### Study design and participants

Data from SELECT-COMPARE (NCT02629159), a phase 3 randomized clinical trial evaluating upadacitinib (UPA) versus adalimumab (ADA) or placebo in patients with moderately-to-severely active RA who were on stable background MTX (≥ 3 months with stable dosage), were used in this post hoc analysis [10, 11].

Patient eligibility criteria have been published elsewhere [10, 11]. Briefly, eligible patients were aged ≥18 years, diagnosed with RA for ≥ 3 months, and were receiving MTX for ≥ 3 months, with a stable dose for ≥ 4 weeks prior to the study start. Patients also had to have a swollen joint count (SJC) and tender joint count (TJC) ≥ 6 at screening and baseline and no prior exposure to a Janus kinase (JAK) inhibitor or ADA. Patients with prior exposure to a bDMARD could be included if they had ≤ 3 months’ exposure and/or discontinued the bDMARD due to intolerance; patients with an inadequate response to a bDMARD were excluded.

Data from patients receiving UPA or ADA for up to 5 years were pooled for the analysis; patients receiving placebo were excluded. Patients included in this analysis were classified as having achieved rapid remission if they achieved a Disease Activity Score 28 with C-reactive protein (DAS28-CRP) < 2.6 at Week 12.

### Outcomes

This analysis assessed the association between: (1) achievement of rapid remission (DAS28-CRP < 2.6 at Week 12); (2) time to achieving remission; and (3) time spent in sustained remission with the mean difference from baseline in a variety of PROs, the odds of achieving minimal clinically important differences (MCIDs) in PROs, and the odds of achieving normative values for CRO/PROs.

The PROs evaluated were: pain measured by 100 mm visual analog scale (VAS), physical function (measured using Health Assessment Questionnaire – Disability Index [HAQ-DI]), quality of life (measured using the Short Form-36 Physical and Mental Component Summary scores [SF-36 PCS and MCS, respectively]), fatigue (measured using Functional Assessment of Chronic Illnesses Therapy-Fatigue [FACIT-Fatigue]), and Patient’s Global Assessment (PGA). CROs included the Physician’s Global Assessment (PhGA), SJC28, and TJC28. MCIDs and normative values for each outcome are listed in Supplementary Table 1.

### Statistical analysis of data

Baseline demographics are reported descriptively. Data are reported as mean change difference with standard deviation at Weeks 26, 48, 256, and 252. Adjusted odds ratios (aOR) with 95% confidence intervals (CI) at Weeks 26, 28, 156, and 252 were reported for the association between achievement of rapid remission and CROs/PROs; for the association between time to achieving remission and time in sustained remission with CROs/PROs, aORs are reported at Week 48. Mean change differences and aORs were calculated using multivariable linear/logistic regression adjusting for age, sex, RA diagnosis duration group, baseline MTX dose group, baseline oral glucocorticoid dose group, baseline DAS28-CRP status, and baseline CRO/PRO score for each outcome evaluated.

For objective 1, outcomes for patients who achieved rapid remission versus those who did not were compared at Weeks 26, 48, 156, and 252.

For objective 2, among patients who achieved remission, time to achieving remission was calculated in months up to Week 48 and used as a continuous variable in the model (the conversion factor used was 1 month = 4.35 weeks). For this analysis, it was not required that patients had to remain in remission (i.e. they could relapse later) but were required to have at least one visit in remission from baseline to Week 48.

For objective 3, time in remission was calculated in months up to Week 48 by a midpoint calculation method. Briefly, at any visit when patients were in remission, the time in remission for that visit was calculated as half of the duration from the earlier visit, plus half of the duration until next visit. Total time in remission was the sum of all duration at the calculation of remission visit based on the midpoint method. The conversion factor used was 1 month = 4.35 weeks.

Nominal *p*-values are reported; a *p*-value < 0.05 was considered statistically significant. Analyses were performed in SAS, Version 9.4 (Cary, NC).

## Results

### Patient demographic and clinical characteristics

Of 885 patients, 28% (247/885) achieved rapid remission at Week 12 (Supplementary Table 2). In general, baseline demographic and clinical characteristics were similar between patients achieving versus not achieving rapid remission. However, patients not achieving rapid remission had numerically higher TJC28, SJC28, and pain scores at baseline. Briefly, patients achieving rapid remission had a mean age of 53.0 ± 12.4 years, with a mean 8.2 ± 7.8 years since RA diagnosis; most patients were female (74.1%) and were receiving concomitant oral glucocorticoids (60.3%).

### Objective 1: the impact of achieving rapid remission on CRO/PROs

Patients who achieved remission at 12 weeks had significantly (*p* < 0.001) greater mean change difference (95% CI) from baseline in all outcomes at Week 26 compared with patients who did not achieve rapid remission (Table 1). The highest numerical differences at Week 26 were for PGA (− 19.7 [− 23.1, − 16.2]), pain VAS (− 19.1 [− 22.5, − 15.7]), and PhGA (− 13.4 [− 16.1, − 10.7]).

**Table 1:** Mean change difference in CRO/PROs between patients who did versus did not achieve rapid remission

Mean change difference^a^ (95% CI)	Week 26 *N* = 388^b^	Week 48 *N* = 436^b^	Week 156 *N* = 417^b^	Week 252 *N* = 387^b^
Pain VAS	−19.1 (− 22.5, − 15.7)***	−16.1 (− 19.5, − 12.6)***	−9.6 (− 13.4, − 5.8)***	−9.1 (− 12.8, − 5.4)***
HAQ-DI	−0.4 (− 0.5, − 0.3)***	−0.4 (− 0.5, − 0.3)***	−0.3 (− 0.4, − 0.2)***	−0.2 (− 0.3, − 0.1)***
SF-36 PCS	6.1 (5.0, 7.2)***	5.7 (4.4, 6.9)***	NC	NC
SF-36 MCS	2.7 (1.3, 4.0)***	1.6 (0.3, 2.9)	NC	NC
FACIT-Fatigue	4.3 (3.0, 5.6)***	4.2 (2.8, 5.6)***	NC	NC
TJC28	−3.2 (− 3.8, − 2.5)***	−2.5 (− 3.1, − 1.9)***	−1.4 (− 2.0, − 0.9)***	−0.6 (− 1.0, − 0.1)*
SJC28	−2.0 (− 2.5, − 1.5)***	−1.3 (− 1.7, − 0.9)***	−0.9 (− 1.3, − 0.5)***	−0.1 (− 0.4, − 0.2)
PGA	−19.7 (− 23.1, − 16.2)***	−17.1 (− 20.7, − 13.5)***	−11.9 (− 15.8, − 8.1)***	−9.5 (− 13.2, − 5.8)***
PhGA	−13.4 (− 16.1, − 10.7)***	−11.3 (− 13.9, − 8.7)***	−5.3 (− 7.7, − 2.9)***	−2.8 (− 4.9, − 0.7)**

*p ≤ 0.05; **p ≤ 0.01; ***p < 0.001

^a^Mean change difference between patients who achieved versus did not achieve rapid remission and 95% CI were evaluated using multivariable linear regression adjusting for age, sex, RA diagnosis duration group, baseline MTX dose group, baseline oral glucocorticoid dose group, baseline DAS28-CRP status, and baseline value of each outcome evaluated. ^b^Total number of patients in remission at the respective timepoint. The number of patients not in remission at Weeks 26, 48, 156, and 252 were 513, 424, 206, and 179, respectively

CI, confidence interval; CRO, clinician-reported outcome; DAS28-CRP, Disease Activity Score 28 with C-reactive protein; FACIT-Fatigue, Functional Assessment of Chronic Illnesses Therapy-Fatigue; HAQ-DI, Health Assessment Questionnaire – Disability Index; MCS, Mental Component Summary; MTX, methotrexate; NC, data not collected at this time point; PCS, Physical Component Summary; PhGA, Physician’s Global Assessment; PGA, Patient’s Global Assessment; PRO, patient-reported outcome; RA, rheumatoid arthritis; SF-36, 36-item Short Form survey; SJC28, swollen joint count in 28 joints; TJC28, tender joint count in 28 joints; VAS, visual analog scale

Patients achieving remission maintained significant mean change differences (*p* ≤ 0.05) in most outcomes up to Week 252.

**Fig. 1:**
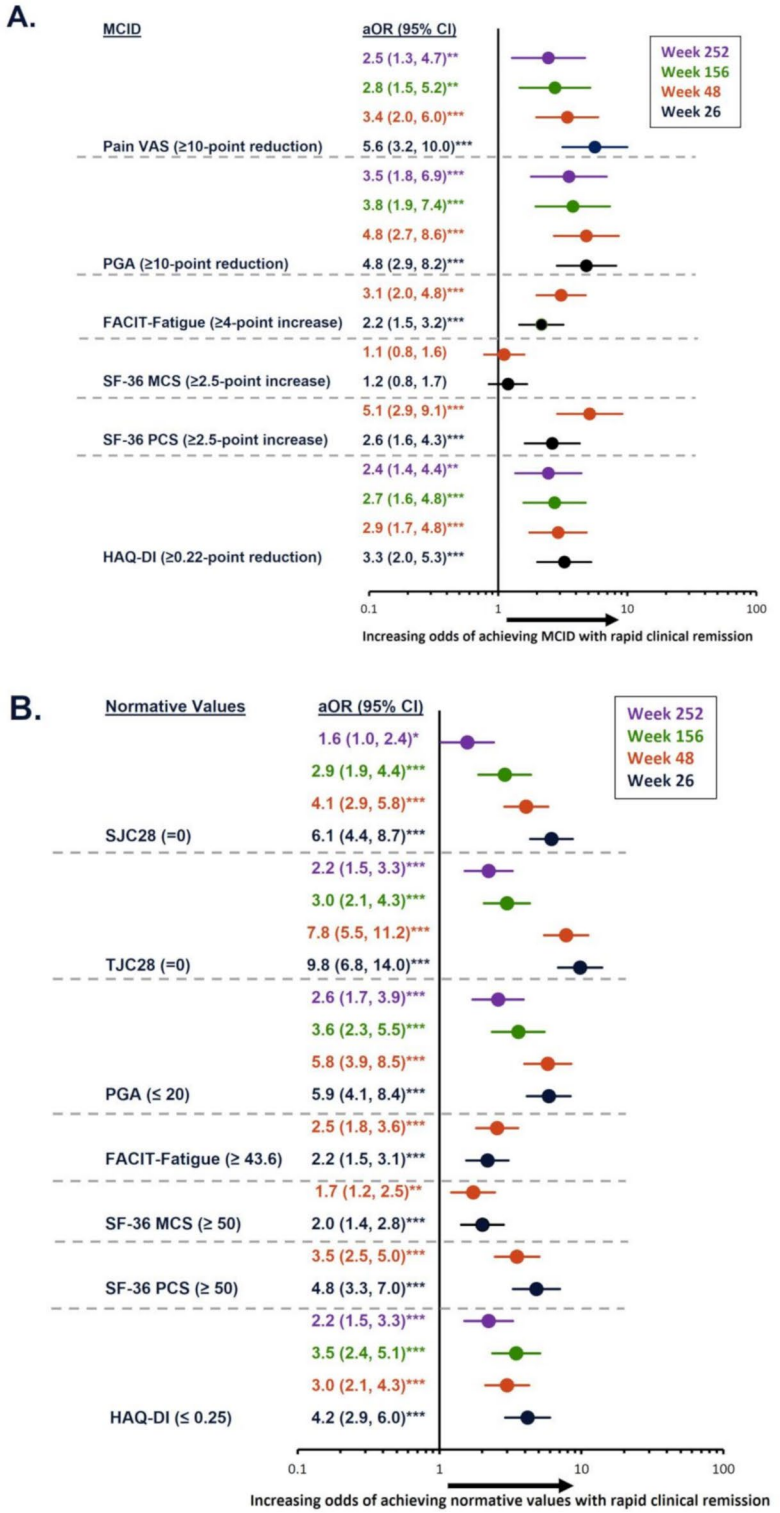
Association of rapid remission with odds of achieving MCID or normative values during 5-year follow-up. *p ≤ 0.05; **p ≤ 0.01; ***p < 0.001. Models were adjusted for the following variables: age, sex, RA diagnosis duration group, baseline MTX dose group, baseline oral glucocorticoid dose group, baseline DAS28-CRP status, and baseline value of each outcome being evaluated. aOR, adjusted odds ratio; CI, confidence interval; DAS28-CRP, Disease Activity Score 28 with C-reactive protein; FACIT-Fatigue, Functional Assessment of Chronic Illnesses Therapy-Fatigue; HAQ-DI, Health Assessment Questionnaire – Disability Index; MCID, minimal clinically important difference; MCS, Mental Component Summary; MTX, methotrexate; PCS, Physical Component Summary; PGA, Patient’s Global Assessment; RA, rheumatoid arthritis; SF-36, 36-item Short Form survey; SJC28, swollen joint count in 28 joints; TJC28, tender joint count in 28 joints; VAS, visual analog scale

Achievement of rapid remission was associated with significantly higher likelihood of achieving MCIDs and/or normative values for the outcomes assessed (Fig. 1). Indeed, relative to those not achieving rapid remission, patients who achieved remission by Week 12 had significantly higher odds of achieving MCID in pain VAS (5.6 [3.2, 10.0]; *p* < 0.001), PGA (4.8 [2.9, 8.2]; *p* < 0.001), FACIT-Fatigue (2.2 [1.5, 3.2]; *p* < 0.001), SF-36 PCS (2.6 [1.6, 4.3]; *p* < 0.001), and HAQ-DI (3.3 [2.0, 5.3]; *p* < 0.001) at Week 26 (Fig. 1A). Odds for achieving MCID remained significantly (*p* < 0.05) higher for pain, PGA, and HAQ-DI for up to 252 weeks. Odds ratios for SF-36 MCS (1.2 [0.8, 1.7]) at Week 26 were numerically higher but did not reach statistical significance.

Similarly, odds for achieving normative values in outcomes at Week 26 were significantly (*p* < 0.001) higher in those achieving versus not achieving rapid remission. Odds of achieving normative values in SJC28, TJC28, PGA, FACIT-Fatigue, SF-36 MCS, SF-36 PCS, and HAQ-DI were 6.1 (4.4, 8.7), 9.8 (6.8, 14.0), 5.9 (4.1, 8.4), 2.2 (1.5, 3.1), 2.0 (1.4, 2.8), 4.8 (3.3, 7.0), and 4.2 (2.9, 6.0), respectively (Fig. 1B); odds remained statistically significant (*p* < 0.05) up to Week 252 for all outcomes (except FACIT-Fatigue and SF-36 PCS/MCS, which were not measured). Overall, among patients achieving rapid remission, the proportion of patients achieving MCIDs or normative values in CRO/PROs was consistent for each outcome over time (Supplementary Fig. 1).

### Objective 2: the impact of time to achieving remission on CRO/PROs

The mean change difference in outcome was assessed based on the time to achieving remission up to Week 48 (Table 2). For every month delay in achieving remission, there was a statistically significant (*p* < 0.001) increase (indicating worsening) in pain VAS (1.8 [1.2, 2.4]), PGA (1.5 [0.9, 2.2]), PhGA (1.0 [0.6, 1.4]), and HAQ-DI (0.04 [0.03, 0.06]) scores at Week 48. Likewise, there were significant decreases in FACIT-Fatigue (–0.3 [–0.6, − 0.1]) and SF-36 PCS (–0.6 [–0.9, − 0.4]) scores, which also indicates worsening outcomes.

**Table 2:** Mean change difference for every month increase in time to remission and time in remission

Mean change difference^a^ (95% CI)	For every month delay in achieving remission	For every additional month spent in remission
	Week 48	Week 48
Pain VAS	1.8 (1.2, 2.4)***	−3.5 (− 3.9, − 3.1)***
HAQ-DI	0.04 (0.03, 0.06)***	−0.08 (− 0.09, − 0.07)***
SF-36 PCS	−0.6 (− 0.9, − 0.4)***	1.2 (1.0, 1.3)***
SF-36 MCS	0.03 (− 0.2, 0.3)	0.4 (0.2, 0.5)***
FACIT-Fatigue	−0.3 (− 0.6, − 0.1)**	0.9 (0.7, 1.0)***
TJC28	0.2 (0.1, 0.2)***	−0.6 (− 0.6, − 0.5)***
SJC28	0.1 (0.0, 0.1)**	−0.3 (− 0.4, − 0.3)***
PGA	1.5 (0.9, 2.2)***	−3.6 (− 4.0, − 3.2)***
PhGA	1.0 (0.6, 1.4)***	−2.3 (− 2.5, − 2.0)***

*p ≤ 0.05; **p ≤ 0.01; ***p < 0.001

^a^Mean change difference between patients for every month delay in achieving remission and every month spent in remission and 95% CI were evaluated using multivariable linear regression adjusting for age, sex, RA diagnosis duration group, baseline MTX dose group, baseline oral glucocorticoid dose group, baseline DAS28-CRP status, and baseline value of each outcome evaluated

CI, confidence interval; CRO, clinician-reported outcome; DAS28-CRP, Disease Activity Score 28 with C-reactive protein; FACIT-Fatigue, Functional Assessment of Chronic Illnesses Therapy-Fatigue; HAQ-DI, Health Assessment Questionnaire – Disability Index; MCS, Mental Component Summary; MTX, methotrexate; NC, data not collected at this time point; PCS, Physical Component Summary; PGA, Patient’s Global Assessment; PhGA, Physician’s Global Assessment; PRO, patient-reported outcome; RA, rheumatoid arthritis; SF-36, 36-item Short Form survey; SJC28, swollen joint count in 28 joints; TJC28, tender joint count in 28 joints; VAS, visual analog scale

**Fig. 2:**
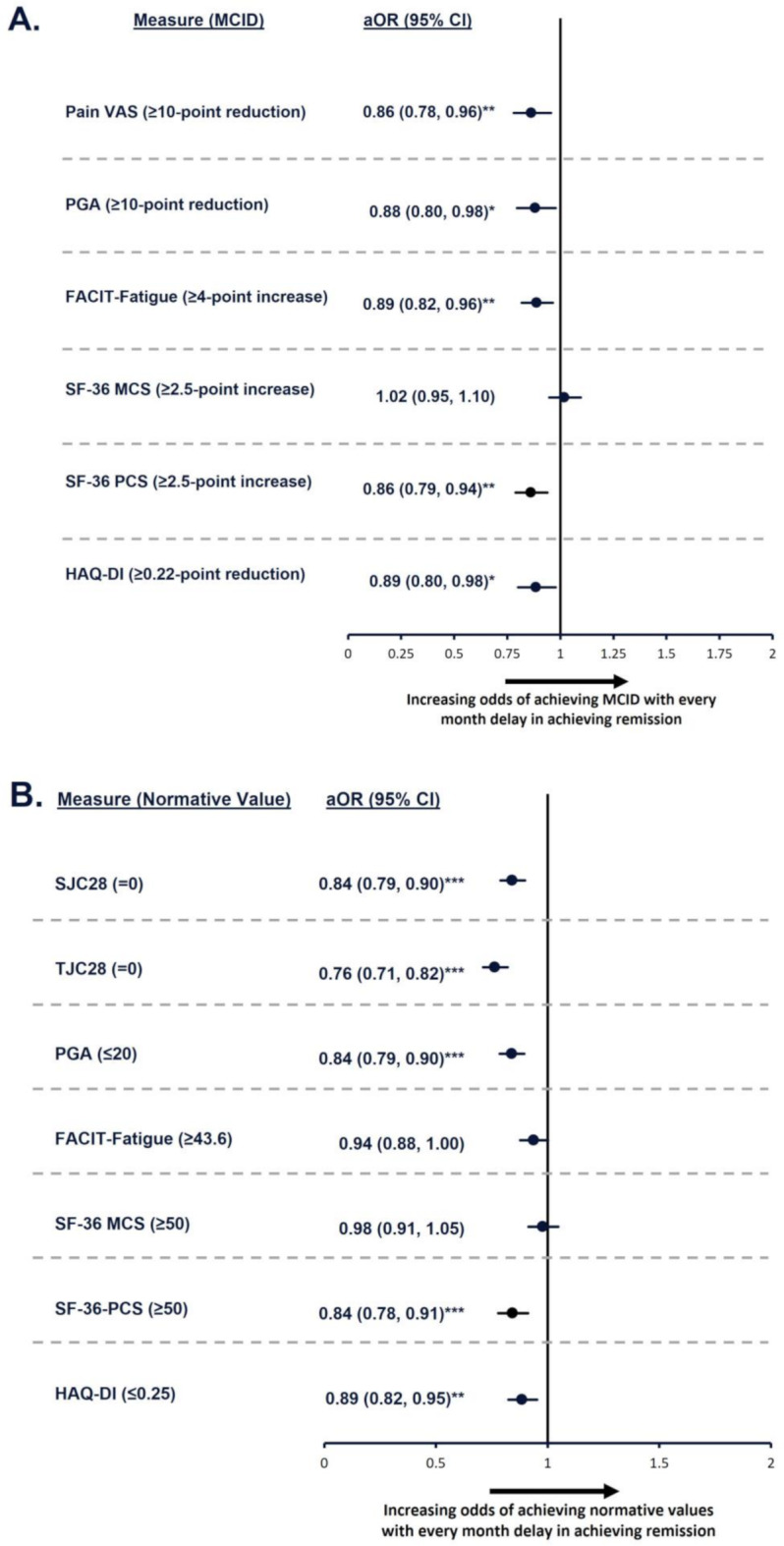
Odds of achieving MCID or normative values for every month delay in achieving remission. *p ≤ 0.05; **p ≤ 0.01; ***p < 0.001. Models were adjusted for the following variables: age, sex, RA diagnosis duration group, baseline MTX dose group, baseline oral glucocorticoid dose group, baseline DAS28-CRP status, and baseline value of each outcome being evaluated. aOR, adjusted odds ratio; CI, confidence interval; DAS28-CRP, Disease Activity Score 28 with C-reactive protein; FACIT-Fatigue, Functional Assessment of Chronic Illnesses Therapy-Fatigue; HAQ-DI, Health Assessment Questionnaire – Disability Index; MCID, minimal clinically important difference; MCS, Mental Component Summary; MTX, methotrexate; PCS, Physical Component Summary; PGA, Patient’s Global Assessment; RA, rheumatoid arthritis; SF-36, 36-item Short Form survey; SJC28, swollen joint count in 28 joints; TJC28, tender joint count in 28 joints; VAS, visual analog scale

For every 1-month delay in achieving remission, odds of achieving MCIDs were reduced by 14% for pain VAS and SF-36 PCS, 12% for PGA, and 11% for FACIT-Fatigue and HAQ-DI (all *p* < 0.05; Fig. 2A). Similarly, odds of achieving normative values in PROs were also impacted by the time to achieving remission (Fig. 2B). Likelihood of achieving normative values decreased by up to 24% (for TJC28; *p* < 0.001), with a mean reduction of 16% (for SJC28, PGA, SF-36 PCS, HAQ-DI; all *p* ≤ 0.01) for every month delay in achieving remission.

### Objective 3: the impact of time in sustained remission on CRO/PROs

**Fig. 3:**
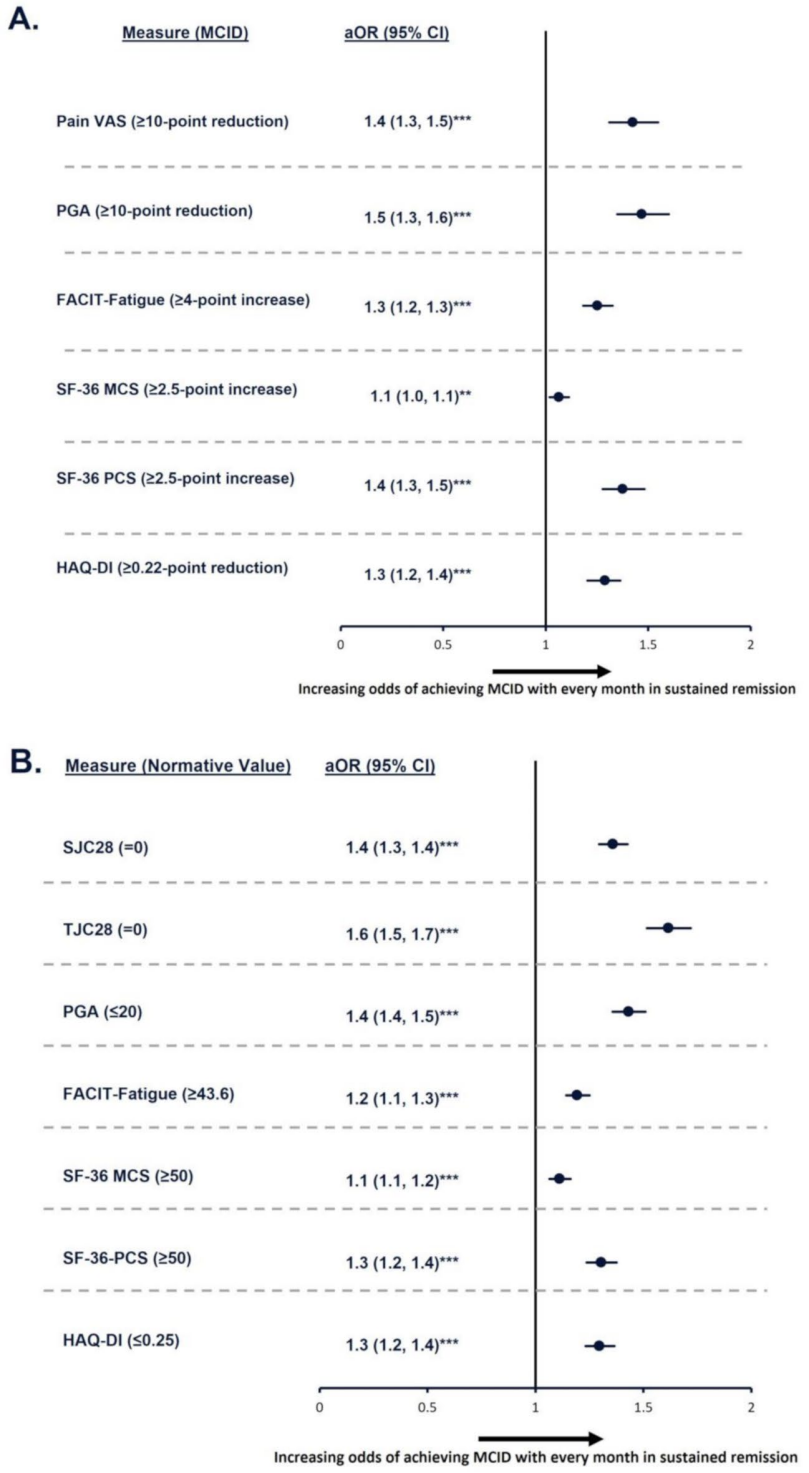
Odds of achieving MCID or normative values for every month in sustained remission at 48 weeks. *p ≤ 0.05; **p ≤ 0.01; ***p < 0.001. Models were adjusted for the following variables: age, sex, RA diagnosis duration group, baseline MTX dose group, baseline oral glucocorticoid dose group, baseline DAS28-CRP status, and baseline value of each outcome being evaluated. aOR, adjusted odds ratio; CI, confidence interval; DAS28-CRP, Disease Activity Score 28 with C-reactive protein; FACIT-Fatigue, Functional Assessment of Chronic Illnesses Therapy-Fatigue; HAQ-DI, Health Assessment Questionnaire – Disability Index; MCID, minimal clinically important difference; MCS, Mental Component Summary; MTX, methotrexate; PCS, Physical Component Summary; PGA, Patient’s Global Assessment; RA, rheumatoid arthritis; SF-36, 36-item Short Form survey; SJC28, swollen joint count in 28 joints; TJC28, tender joint count in 28 joints; VAS, visual analog scale

For every additional month in remission up to Week 48, patients experienced significant (*p* < 0.001) improvements in all outcomes assessed (Table 2) and significantly (*p* ≤ 0.05) greater odds of reaching MCIDs for pain VAS (1.4 [1.3, 1.5]), PGA (1.5 [1.3, 1.6]), FACIT-Fatigue (1.3 [1.2, 1.3]), SF-36 MCS (1.1 [1.0, 1.1]), SF-36 PCS (1.4 [1.3, 1.5]), and HAQ-DI (1.3 [1.2, 1.4]; Fig. 3A). Odds of achieving normative values were 60% higher for each additional month in remission for TJC28 (1.6 [1.5, 1.7]), 40% higher for SJC (1.4 [1.3, 1.4]) and PGA (1.4 [1.4, 1.5]), and 30% higher for SF-36 PCS (1.3 [1.2, 1.4]) and HAQ-DI (1.3 [1.2, 1.4]; Fig. 3B). For all outcomes, odds of achieving normative values were highly statistically significant (*p* < 0.001).

## Discussion

These data further confirm existing evidence of the impact of achieving rapid and sustained remission on long-term CROs and PROs in patients with moderately-to-severely active RA and illustrate the magnitude of achievable outcomes with b/tsDMARDs in patients with an inadequate response to MTX treatment. Patients who achieved rapid remission had significantly higher odds of achieving MCIDs and normative values in most PROs as early as Week 26; this was maintained for up to 252 weeks. Similarly, these data demonstrated that, for every month delay in achieving remission, the likelihood of achieving MCIDs or normative values in these PROs was significantly reduced. In contrast, for every additional month a patient was in sustained remission, odds were significantly higher.

This analysis is consistent with previous studies which have shown that achieving remission earlier has a significant impact on clinical outcomes [8, 9]. Indeed, a study in patients with RA receiving MTX with or without certolizumab pegol showed that patients who did not achieve clinical remission after 12 weeks had significantly greater radiographic disease progression at Week 52 than those who achieved remission earlier [8]. Studies have shown, however, that achieving remission also has a significant impact on long-term patient-reported HRQoL and work and activity ability [2–4]. A study among 356 consecutive patients with RA on a heterogenous mix of treatment regimens showed that patients who achieved remission had SF-36 PCS scores of 46.0 (≥ 50 is normative value) compared with a score of 29.8 among patients with moderate-to-high disease activity [3]. Similarly, patients achieving remission reported < 12% impairment in work productivity or activity ability, versus 46% impairment reported by patients with moderate-to-high disease activity [3].

This analysis showed that time to remission had a significant impact on PROs, with those achieving remission earlier reporting better outcomes. This is consistent with an analysis of participants in the tREACH (treatment in the Rotterdam Early Arthritis CoHort) study, which demonstrated that patients who achieve remission in the first year reported better physical functioning and less pain and fatigue in the subsequent years compared with patients not achieving remission in the first year [12]. Moreover, a survey of patients with RA demonstrated that, for patients, the duration of remission, as well as the perception of returning to normal, is a key component of the concept of remission [13].

A Dutch longitudinal study of patients with RA showed that the likelihood of ever achieving sustained remission was significantly reduced with increasing time to remission, further highlighting the importance of rapid remission in long-term outcomes of patients with RA [7]. Moreover, a separate study of patients with RA receiving tumor necrosis factor inhibitors with or without MTX showed that the disease activity observed within the first 3 months of treatment was significantly related to disease activity at 1 year [9]. Compared with those who do not achieve remission, patients with RA achieving remission also had up to 52% lower direct medical costs and up to 75% lower indirect costs [4]. Thus, achieving remission also reduces the economic burden of disease.

This study used data from the phase 3 SELECT-COMPARE trial. Hence, the quality and completeness of the data are better compared to an observational study. The open-label extension phase of the study also provides data for up to 5 years, making it possible to examine the associations between the variables of interest over a long period of time. This is the first study to evaluate the association between rapid and sustained remission and CRO/PROs in patients with RA using advanced therapies. Due to the specific inclusion and exclusion criteria of the clinical trial program, the patients included in this study may not represent the overall population of patients with RA and therefore the results of this study may not be generalizable to all patients with RA. However, the intention of this analysis was to assess the impact of achieving rapid remission independent of active drug, thus allowing the results to be more generalizable to a larger population of patients with RA. This is a post hoc analysis of two groups of patients, thus the characteristics of patients achieving remission versus those not achieving remission may not be uniformly distributed. Despite controlling for patients’ baseline remission status and the baseline score of the outcome being evaluated, confounders may still exist.

## Conclusions

This post hoc analysis demonstrated that patients with moderately-to-severely active RA who achieved rapid remission with UPA or ADA, compared with those who did not, had less subsequent pain and fatigue. Moreover, patients achieving rapid remission reported better physical function and quality of life for up to 5 years versus patients who did not reach rapid remission. Shorter time to achieving remission, as well as longer time spent in in sustained remission, also correlated with sustained improvement in CRO/PROs. Overall, these data highlight the benefits of effective therapy and achieving rapid remission on long-term disease-related burden in patients with moderately-to-severely active RA.

## Electronic supplementary material

Below is the link to the electronic supplementary material.

Supplementary Material 1

## Data Availability

The datasets analyzed for the current study are available from the corresponding author on reasonable request.

## Abbreviations

(b/ts)DMARD: Biologic or targeted synthetic DMARD
ADA: Adalimumab
aOR: Adjusted odds ratio
bDMARD: Biologic DMARD
CI: Confidence interval
CRO: Clinician-reported outcome
CRP: C-reactive protein
DAS28: Disease Activity Score 28
DMARD: Disease-modifying antirheumatic drug
FACIT-Fatigue: Functional Assessment of Chronic Illnesses Therapy-Fatigue
HAQ-DI: Health Assessment Questionnaire – Disability Index
HRQoL: Health-related quality of life
JAK: Janus kinase
MCID: Minimal clinically important difference
MCS: Mental Component Summary
MTX: Methotrexate
NA: Not applicable
NC: Data not collected at this time point
PCS: Physical Component Summary
PGA: Patient’s Global Assessment
PhGA: Physician’s Global Assessment
PRO: Patient-reported outcome
RA: Rheumatoid arthritis
SD: Standard deviation
SF-36: 36-item Short Form survey
SJC(28): Swollen joint count (in 28 joints)
TJC(28): Tender joint count (in 28 joints)
tREACH: Treatment in the Rotterdam Early Arthritis CoHort
UPA: Upadacitinib
VAS: Visual analog scale
