# Supplementary material for: Association between rapid and sustained remission and clinician- and patient-reported outcomes in patients with rheumatoid arthritis: post hoc analysis of data from the SELECT-COMPARE study
Source: Arthritis Res Ther. 2025 Jun 13;27:123. doi: 10.1186/s13075-025-03580-1 (PMC12164154; doi:10.1186/s13075-025-03580-1)
Supplement: Supplementary file 1 — Supplementary Material 1 [file 13075_2025_3580_MOESM1_ESM.docx]

**Association between rapid and sustained remission and clinician- and patient-reported outcomes in patients with rheumatoid arthritis: post hoc analysis of data from the SELECT-COMPARE study**

**Running title:** Value of rapid and sustained remission in rheumatoid arthritis

Authors: Laure Gossec^1,2^, Jayesh Patel^3^, Aditi Kadakia^3^, Siran Fang^3^, Yi Peng^3^, Sander Strengholt^3^, Peter C. Taylor^4^ and Andrew Östör^5^

Affiliations: ^1^Sorbonne Université, Institut Pierre Louis d'Epidémiologie et de Santé Publique, Paris, France; ^2^AP-HP, Pitié Salpêtrière Hospital, Paris, France; ^3^AbbVie Inc, North Chicago, IL, USA; ^4^Botnar Research Centre, University of Oxford, Oxford, UK, ^5^Monash University and Emeritus Research Melbourne & ANU Canberra, Australia.

Corresponding Author: Aditi Kadakia

Dept GMH1, Blg ABV1-4SE-108-01

26525 North Riverwoods Blvd.

Mettawa, IL 60045

Phone: 781-759-3799

Email: [aditi.kadakia@abbvie.com](mailto:aditi.kadakia@abbvie.com)

Target Journal: *Arthritis Research and Therapy*

Figure/Table count: 2 tables, 3 figures (no limit)

Word Count: 2545 (no limit)

# Supplementary material

Supplementary Table 1 MCID and normative values for outcomes assessed^1^

| PRO | Range | MCID | Normative value |
| --- | --- | --- | --- |
| Pain (VAS) | 0–100 mm | ≥10 mm reduction | NA |
| HAQ-DI | 0–3 | ≥0.22-point reduction | ≤0.25 points |
| SF-36 PCS/MCS | 0–100 | ≥2.5-point increase | ≥50 points |
| FACIT-Fatigue | 0–52 | ≥4.0-point increase | ≥43.6 points |
| PGA | 0–100% | ≥10-point reduction | ≤20 points |
| SJC28/TJC28 | 0–28 | NA | 0 |

1. Strand V, Tundia N, Bergman M, et al. Upadacitinib improves patient-reported outcomes vs placebo or adalimumab in patients with rheumatoid arthritis: results from SELECT-COMPARE. Rheumatology (Oxford). 2021. doi:10.1093/rheumatology/keab158.

FACIT-Fatigue, Functional Assessment of Chronic Illness Therapy – Fatigue; HAQ-DI, Health Assessment Questionnaire – Disability Index; MCID, minimal clinically importance difference; MCS, Mental Component Summary; NA, not applicable; PCS, Physical Component Summary; PGA, Patient’s Global Assessment; PRO, patient-reported outcome; SF-36, 36-item Short Form survey; SJC28, swollen joint count in 28 joints; TJC28, tender joint count in 28 joints; VAS, visual analog scale.

Supplementary Table 2 Baseline demographic and clinical characteristics

| Characteristic | Patients achieving rapid remission  N=247 | Patients **not** achieving rapid remission  N=638 | *P*-value |
| --- | --- | --- | --- |
| **Age** [years], mean ± SD | 53.0 ± 12.4 | 54.5 ± 11.5 | .097 |
| **Female sex**, n (%) | 183 (74.1) | 520 (81.5) | .014 |
| **Duration of RA diagnosis** [years], mean ± SD | 8.2 ± 7.8 | 8.3 ± 8.2 | .953 |
| **MTX dose** [mg], mean ± SD | 17.3 ± 4.3 | 17.0 ± 4.0 | .417 |
| **Concomitant oral glucocorticoid**,^a^ n (%)  Daily dose [mg], mean ± SD | 149 (60.3)  6.0 ± 2.2 | 407 (63.8)  6.7 ± 2.5 | .001 |
| **In DAS28-CRP remission**,^a^ n (%)  Yes  No | 1 (0.4)  245 (99.6) | 0 (0)  634 (100) | .108 |
| **DAS28-CRP Score,** mean ± SD  At Baseline | 5.4 ± 1.0 | 6.0 ± 0.9 | <.001 |
| **TJC28**, mean ± SD | 12.8 ± 6.5 | 15.9 ± 6.8 | <.001 |
| **SJC28**, mean ± SD | 10.3 ± 4.7 | 12.0 ± 7.9 | <.001 |
| **SF-36 PCS**, mean ± SD | 35.1 ± 7.9 | 31.3 ± 6.7 | <.001 |
| **SF-36 MCS**, mean ± SD | 45.1 ± 10.6 | 42.0 ± 10.6 | <.001 |
| **HAQ-DI**, mean ± SD | 1.4 ± 0.7 | 1.7 ± 0.6 | <.001 |
| **Pain** [VAS], mean ± SD | 59.1 ± 23.5 | 68.3 ± 19.3 | <.001 |
| **FACIT-Fatigue**, mean ± SD | 30.2 ± 11.3 | 25.2 ± 10.9 | <.001 |
| **PGA**, mean ± SD | 60.5 ± 18.0 | 67.1 ± 16.8 | <.001 |
| **PhGA**, mean ± SD | 57.3 ± 24.3 | 67.4 ± 19.9 | <.001 |

^a^Patients with missing baseline values were excluded from calculations. DAS28-CRP, Disease Activity Score 28 with C-reactive protein; FACIT-Fatigue, Functional Assessment of Chronic Illnesses Therapy-Fatigue; HAQ-DI, Health Assessment Questionnaire – Disability Index; MCS, Mental Component Summary; MTX, methotrexate; PCS, Physical Component Summary; PGA, Patient’s Global Assessment; PhGA, Physician’s Global Assessment; RA, rheumatoid arthritis; SD, standard deviation; SF-36, 36-item Short Form survey; SJC28, swollen joint count in 28 joints; TJC28, tender joint count in 28 joints; VAS, visual analog scale.

Supplementary Fig. 1 Proportion of patients achieving MCID or normative values in CRO/PROs over time^a^


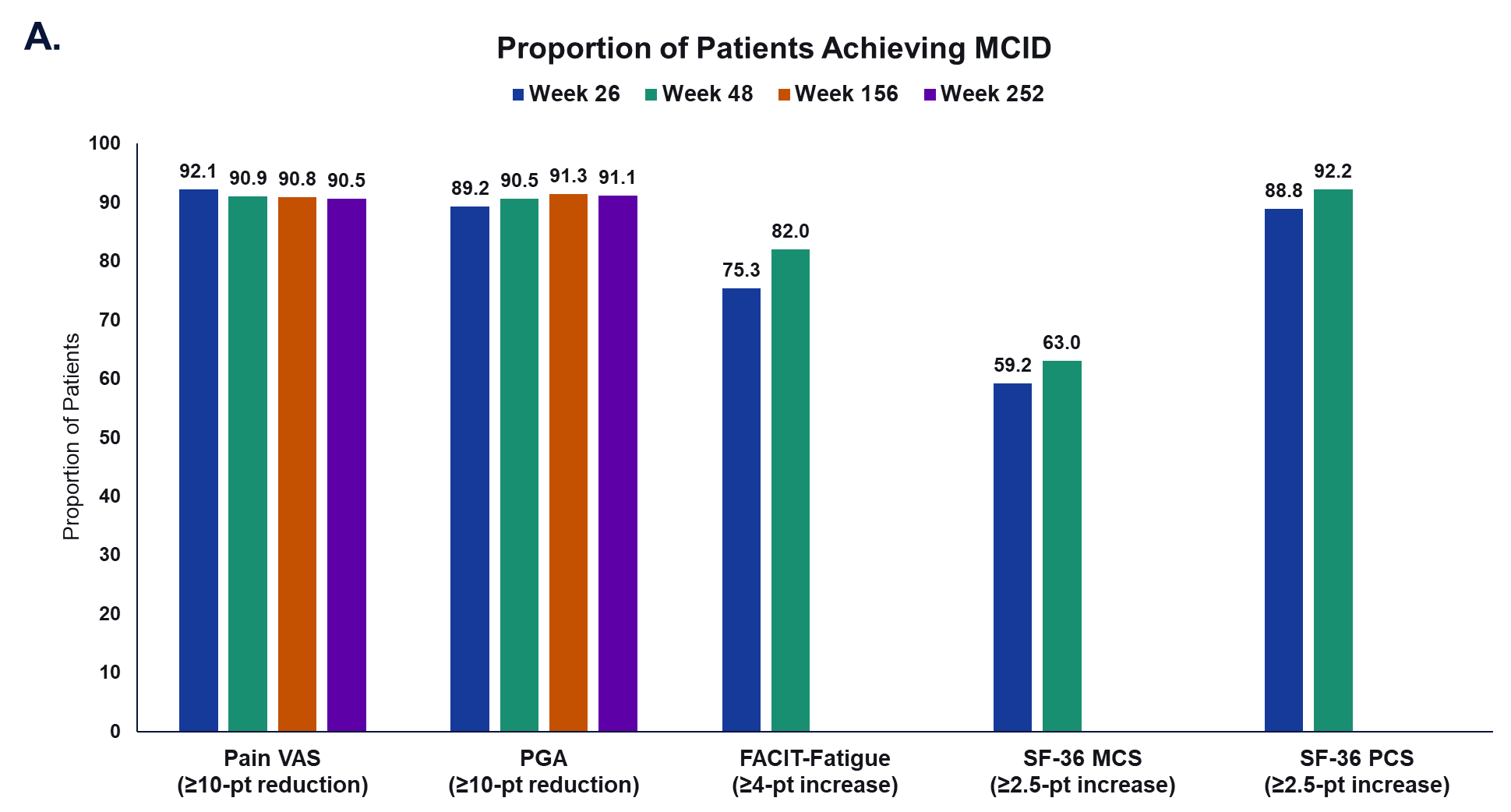


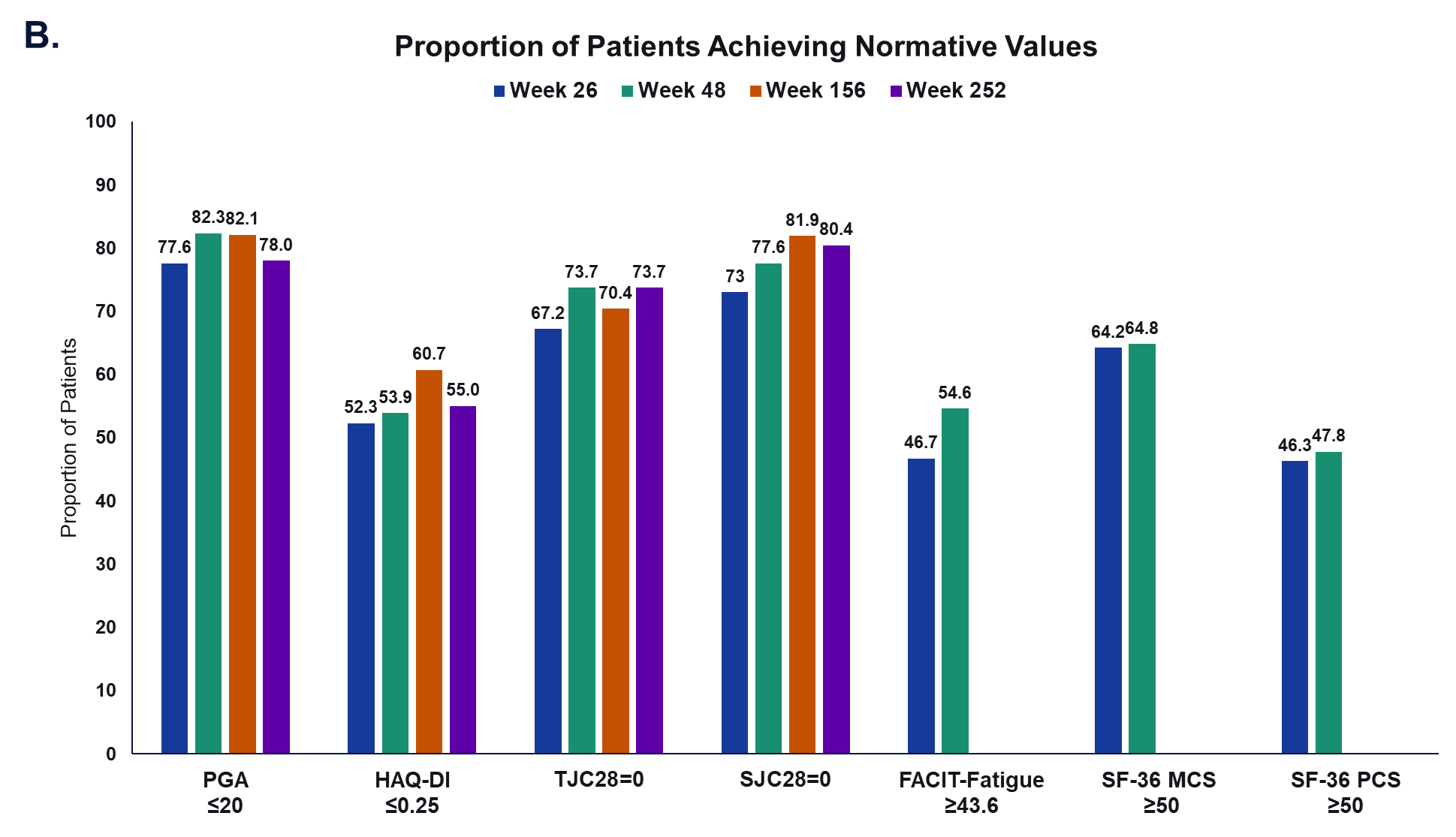


^a^Among patients who achieved rapid remission. CRO, clinician-reported outcome; FACIT-Fatigue, Functional Assessment of Chronic Illnesses Therapy-Fatigue; HAQ-DI, Health Assessment Questionnaire – Disability Index; MCID, minimal clinically important difference; MCS, Mental Component Summary; PCS, Physical Component Summary; PRO, patient-reported outcome; PGA, Patient’s Global Assessment; SF-36, 36-item Short Form survey; SJC28, swollen joint count in 28 joints; TJC28, tender joint count in 28 joints; VAS, visual analog scale.
